# Assessment of Clinical Parameters of Dental Anxiety during Noninvasive Treatments in Dentistry

**DOI:** 10.3390/ijerph191711141

**Published:** 2022-09-05

**Authors:** Gabriela Gil-Abando, Paula Medina, Carolina Signorini, Elisabeth Casañas, Natalia Navarrete, Marta Muñoz-Corcuera

**Affiliations:** Department of Clinical Dentistry, Faculty of Biomedical and Health Sciences, Universidad Europea de Madrid, 28005 Madrid, Spain

**Keywords:** dental anxiety, blood pressure, heart rate, Corah Dental Anxiety Scale

## Abstract

Dental anxiety is a matter of interest for the dentist since an anxious patient is a potential source of complications in the dental office. The main objectives of this study are to describe the correlation between dental anxiety levels and the values of physiological parameters related to dental anxiety and to study the evolution of blood pressure and heart rate over time during noninvasive dental treatments, i.e., not requiring local anesthesia. A descriptive, longitudinal, and prospective observational study was designed. The study population consisted of 200 patients who attended a university clinic for dental treatment without local anesthesia. The patients were asked to complete the Corah Dental Anxiety Scale. Afterward, blood pressure and heart rate were measured by means of a digital sphygmomanometer. Blood pressure and heart rate were taken throughout the procedure on four occasions. Most of the patients showed mild dental anxiety (5 [IQR: 3] points on Corah Dental Anxiety Scale). Significant but weak correlations were found between the level of dental anxiety and heart rate (Spearman rho: 0.166 and 0.176; *p* = 0.019 and 0.013; 3 min before and after treatment, respectively), as well as between the level of dental anxiety and the duration of treatment (Spearman rho: 0.191 3 min; *p* = 0.007). As for the evolution of physiological parameters, all patients showed a progressive decrease in values at different time points during treatment. When the types of treatment were evaluated separately, it was observed that there were statistically significant differences between them with respect to the level of dental anxiety (*p* = 0.006).

## 1. Introduction

Anxiety disorders are a diversified group of mental health disorders characterized by hyperactivity, excessive fear, and worry [1].

When undergoing dental treatment, dental anxiety is a factor to be taken into account since an anxious patient is a potential source of complications in the dental office. Anxiety disorders are also associated with elevated cardiovascular risk factors such as arterial hypertension, as well as a higher rate of cardiovascular disease and premature mortality [2].

Every hypertensive patient should be checked prior to dental care, and their blood pressure (BP) values should be recorded before starting any treatment. Routine measurement of BP could reduce the risk of cardiovascular accidents and acute complications during dental treatment [3].

It is an evident fact that visits to the dentist are stressful for patients, no matter how simple the treatment or intervention is [4]. We consider it important to study dental anxiety in all types of dental interventions, given the potential impact that it can have on professionals and their patients.

Several studies in our field have reported low or moderate anxiety values [4,5], with higher scores in women [5,6]. The data according to age are more variable since some authors have reported higher levels of anxiety among young people [6], and other authors have reported higher levels in older patients [5]. The stress associated with dental anxiety produces an increase in heart rate (HR) and respiratory rate, as well as an increase in BP, which in a hypertensive patient could lead to a hypertensive crisis. Along these lines, it has been published the relationship between dental anxiety and diastolic blood pressure (DBP) and HR values [7]. Other authors [8,9] have studied the existing differences between hypertensive and normotensive patients in terms of dental anxiety, BP, and HR, without finding significant differences between them, although pointing out that hypertensive patients may be more affected by dental anxiety in terms of the physiological parameters related to it.

The present study aimed to evaluate dental anxiety from two points of view: the dental anxiety levels and physiological parameters for evaluating the level of dental anxiety.

The **hypothesis** of this study is that the patient’s dental anxiety levels are related to BP values. A significant correlation (r > 0.2) is expected between the Corah Dental Anxiety Scale score and the systolic blood pressure (SBP) on arrival at the office.

The **main objectives** of this study are to describe the correlation between anxiety levels and the values of physiological parameters related to dental anxiety and to study the evolution of blood pressure and heart rate over time during noninvasive dental treatments, i.e., not requiring local anesthesia. Additionally, differences between hypertensive and normotensive patients, between patients with different anxiety levels, and according to sex, age, and type of treatment carried out.

## 2. Materials and Methods

A descriptive, longitudinal, prospective, observational study was designed. The study protocol was approved by the ethics committee of the European University of Madrid on 14 October 2020. All patients signed informed consent for participation in the study.

### 2.1. Study Population

The study population consisted of patients who attended the university clinic for noninvasive dental treatment between October 2020 and June 2021.

The inclusion criteria were patients over 18 years of age, ASA I and II patients who voluntarily participated in the study and who signed the informed consent form.

Pregnant patients were excluded.

Patients were included sequentially, as they came to the university clinic to undergo dental treatments not requiring local anesthesia. 

*Sample size calculation*: It was calculated that the inclusion of 200 patients would be sufficient to estimate, with a confidence level of 95% and a statistical power of 80%, correlation coefficients between dental anxiety and SBP on arrival at the clinic greater than or equal to 0.2.

### 2.2. Procedure

The patient came in for the necessary dental treatment. He/she was given the information sheet about the study and signed the informed consent in order to participate.

Once this had been conducted, the patient was asked to complete the Corah Dental Anxiety Scale. As a tool for measuring dental anxiety, the Corah Dental Anxiety Scale [10], a questionnaire that has demonstrated high efficacy and accuracy in its results, is frequently used. It is a short, brief and reliable survey consisting of 4 questions with 5 possible answers (A, B, C, D, and E). Each of them is assigned a numerical value (1, 2, 3, 3, 4, or 5), which is then added at the end and provides an assessment of the dental anxiety level. A patient with less than 9 points would suffer from mild dental anxiety, between 9 and 12 points of moderate dental anxiety, between 13 and 14 points of high dental anxiety, and more than 15 points indicates severe dental anxiety [11,12,13].

Next, BP and HR measurements were recorded by means of an OMRON M6 digital sphygmomanometer. Four measurements were taken (1) when the patient arrived at the dental office, (2) three minutes before starting the treatment, (3) three minutes after starting the treatment, and (4) once the treatment finished.

The treatments performed were first visits, check-ups, prosthetic treatments (taking impressions and structure try-ins), periodontal studies, and prophylaxis.

### 2.3. Variables

Different variables were collected: sex and age of the patient, whether they were hypertensive or normotensive, the type of treatment to be performed and its duration, the score on the Corah Dental Anxiety Scale, and four measurements of BP and HR.

### 2.4. Statistical Analysis

A descriptive study was performed using the mean ± standard deviation or median and interquartile range [IQR: Q3–Q1] according to the normality of quantitative variables. Qualitative variables were described by absolute (n) and relative (%) frequency.

Student’s *t*-tests for independent samples or Mann–Whitney U tests were used for comparison of means between the groups of normotensive and hypertensive patients, as well as between sexes and age groups; and also to study possible differences in the change in SBP, DBP, and HR between each time point between normotensive and hypertensive patients. Mann Whitney U tests were used for comparison of SBP, DBP, and HR between patients with low dental anxiety (Corah test < 9 points) and patients with moderate-high dental anxiety (Corah test ≥ 9 points).

Repeated measures ANOVA or Friedman’s tests were used to study the evolution of SBP, DBP, and HR over time.

To study the correlation between the Corah Dental Anxiety Scale score and the physiological parameters, as well as their evolution and the duration of the procedure, Pearson or Spearman coefficients and their significance were calculated by applying the Bonferroni correction.

A significance level of 5% was established in all the analyses, and the STATA IC version 14.2 program (StataCorp, LLC. College Station, TX, USA) was used.

## 3. Results

### 3.1. Descriptive Analysis

A total of 200 patients were included in the study. The age ranged from 18 to 82 years, with an average of 48.17 ± 16.26 years and a median of 52 [IQR 24.5] years.

As for the distribution of the sample by sex, women predominated, accounting for 62.5% versus 36.5% of men. Of the total number of patients included in the study, 24% were hypertensive according to their medical history.

The average time taken to perform the treatments was 70.34 ± 31.11 min, with a minimum of 7 min and a maximum of 183 min.

Most of the patients were subjected to mild dental anxiety (86.5%), 10% presented moderate dental anxiety, and very few had high or severe dental anxiety (3.5%). The median score on the Corah Dental Anxiety Scale was 5 [IQR 3] points. The minimum value obtained was 4, and the maximum value was 19 points.

The values of SBP, DBP, and HR at the four measurement points for the whole sample are shown in Table 1.

### 3.2. Evolution of Physiological Parameters

**Figure 1:**
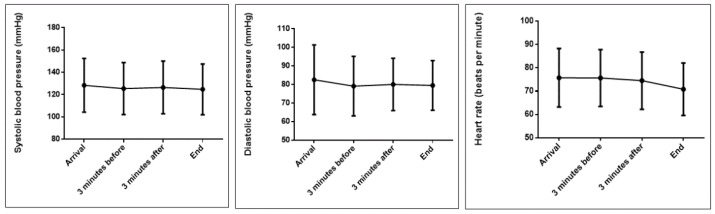
Evolution of physiological parameters in patients as a whole.

As for the evolution of physiological parameters over time, Figure 1 shows how the SBP, DBP, and HR evolved in the group of patients throughout the treatment. The highest measurements were those recorded on arrival at the clinic. Subsequently, they decreased until the end. The repeated measures ANOVA test with a Greenhouse–Geisser correction determined that the SBP, DBP, and HR differed between time points (*p* = 0.001, *p* = 0.007, and *p* < 0.001, respectively).

### 3.3. Comparison between Hypertensive and Normotensive Patients

The proportion of men was significantly higher among hypertensive patients than in normotensive patients (69.08% vs. 41.67%; *p* = 0.001). The median age was significantly higher in hypertensive patients with respect to normotensive patients (59 [17.50] vs. 46.50 [23], *p* ˂ 0.001).

Significant differences were found in the values of SBP and DBP, which were higher in the hypertensive group at all measurement points. HR levels were similar in both groups (Table 2).

The Corah Dental Anxiety Scale score was similar in both groups, and no statistically significant differences were found between them. The median for hypertensives was 5 [3] and for normotensives 4 [3.50] (*p* = 0.998).

In both groups, the repeated measures ANOVA test with a Greenhouse–Geisser correction determined that the SBP values were lower at completion than at arrival, with the change being statistically significant (*p* = 0.033 for normotensives and *p* = 0.022 for hypertensives).

As for DBP, there were statistically significant differences at arrival and at the time of completion in normotensive patients (*p* = 0.012) but not in hypertensive patients (*p* = 0.093).

**Figure 2:**
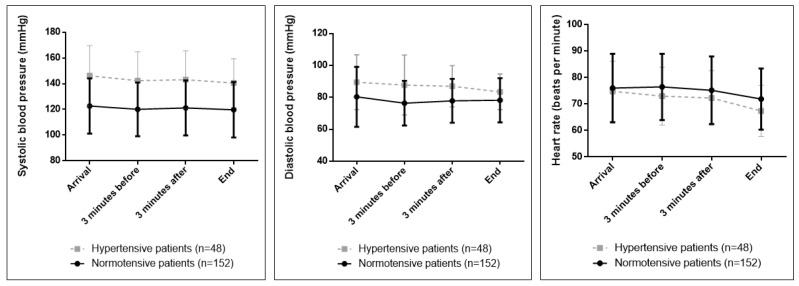
Evolution of physiological parameters in hypertensives and normotensives.

HR recordings were lower in the 4 points recorded in hypertensives than in normotensives. In both groups, the values were significantly lower at the end with respect to all measurement points (*p* < 0.001 in normotensives and *p* < 0.001 in hypertensives) (Figure 2).

When analyzing the differences in the evolution of the parameters between the two groups, it was observed that there were no significant differences in the evolution of SBP, but there were significant differences in the evolution of DBP and HR, with the evolution of these parameters being different between the arrival of the patient and the completion of the treatment between the two groups. DBP and HR decreased more in hypertensives than in normotensives from arrival to the end of treatment.

### 3.4. Correlations

The possible correlations between the level of dental anxiety and SBP, DBP, HR, and treatment duration were studied.

For the patients as a whole and for the normotensive group, significant but weak correlations were found between the level of dental anxiety and HR, as well as between the level of dental anxiety and the duration of treatment. The correlation between dental anxiety and SBP/DBP was not significant. In hypertensive patients, all correlations were not significant (Table 3).

### 3.5. Comparisons by Gender

When comparing men and women, both in the group of patients and when assessing normotensive and hypertensive patients separately, dental anxiety was significantly higher in women. In the set of patients as a whole, SBP and DBP on arrival were significantly higher in men. The data relating to these comparisons are shown in Table 4.

### 3.6. Comparisons by Age

Comparisons were made according to age on the different study variables, dividing the patients into two groups: (1) those above the median and (2) those below the median of 52 years of age. Significant differences were found only in the values of SBP and DBP on arrival, in patients as a whole, and in normotensive patients between those older and younger than 52 years of age. The data are shown in Table 5.

### 3.7. Comparisons by Treatment Type

When comparing the types of treatment performed, in the measurements of physiological parameters on arrival, no significant differences were found between the treatment groups, but there were significant differences in the levels of dental anxiety, with the periodontal studies being those in which the patients had a higher score on the Corah Dental Anxiety Scale. The data are shown in Table 6.

### 3.8. BP and HR Comparisons by Anxiety Level

Finally, the SBP, DBP, and HR values at arrival were compared between patients with different levels of anxiety. For this purpose, patients who registered less than 9 points on the Corah Dental Anxiety Scale were grouped on the one side (86.5%) and those who registered 9 or more points on the said scale on the other side (13.5%). No significant differences were found in terms of SBP or DBP. Significant differences were observed, with the HR being higher in the patients who presented greater dental anxiety. The data are shown in Table 7.

## 4. Discussion

In this study, an attempt was made to see if there was a correlation between the levels of dental anxiety perceived by the patient and their levels of BP and HR in dental treatments without local anesthesia. It seems logical to assume that there is a relationship between the score obtained on the Corah Dental Anxiety Scale, BP, and HR. In spite of this, the results show a very weak significant correlation between the level of dental anxiety and HR. Along the same lines, in the study by Fernández-Aguilar et al. [7], the authors found significant differences between dental anxiety and DBP and HR, and no relationship was found between dental anxiety and SBP.

Positive correlations (although weak) were found between the level of dental anxiety and treatment time, in the patients as a whole and in the normotensive group. The hypertensive patients in this study were less affected in terms of dental anxiety by the duration of treatment than the normotensive patients. Treatments carried out at the university clinic have very long execution times because they are undergraduate students who are beginning to develop their abilities and manual skills. In addition, students transfer their level of dental anxiety about having to perform a treatment for the first time to the patient himself, which is detrimental to both. If the patient knows beforehand that the procedure will be long or uncomfortable, the anxiety level could be higher and may be affected by the type of treatment that the patient was assigned.

The Corah Dental Anxiety Scale was used to assess the patient’s pre-treatment dental anxiety. The study by Alemany-Martínez et al. [14] included patients who underwent surgical extractions of the lower third molars, and women showed higher levels of dental anxiety than men. In this study, women had significantly higher levels of dental anxiety than men. Settineri et al. [15] analyzed the presence of dental anxiety, and it was greater in women than in men during waiting time before anesthesia and during dental preparation. Tarazona et al. [5]. Caltabiano et al. [6] and Hu et al. [16] showed that women suffer greater dental anxiety than men. However, Fernandez-Aguilar et al. [7] found no differences in dental anxiety between men and women. Ferreira Gaona et al. [12], using the Corah Dental Anxiety Scale, showed a low number of patients with extreme dental anxiety, coinciding with our study.

In our study, despite not performing interventions under the effects of local anesthesia, there were patients who presented high levels of dental anxiety, and in the case of hypertensive patients, this dental anxiety should be controlled to reduce the risk of the appearance of possible complications in the cabinet. Each time the SBP increases by 20 mmHg or the DBP increases by 10 mmHg, the patient’s risk of a cardiovascular incident double [17].

In this study, a decrease in BP and HR values was recorded throughout the treatment, with significant variation between the different measurement points. In the study by Alemany-Martínez et al. [14], variations in BP and HR during surgical extraction of molars were within normal limits, but a rise followed by a subsequent decline was observed. Nicolosi et al. [18] performed five determinations of BP and HR throughout the dental treatment. The record-taking was very similar to that performed for this work. These authors observed, similarly to what occurred in this study, higher values of SBP, DBP, and HR in hypertensive patients than in normotensive patients. As published by Alemany-Martínez et al. [14], they observed an increase in values at the central measurement point followed by a decrease. In this study, this was not the case; a gradual decrease was observed from the arrival point to the end of the procedure. Once again, the differences observed are given by the methodological difference and by the fact that we are not analyzing surgical procedures.

Carrasco et al. [19] conducted a study on 30 normotensive and hypertensive individuals. They recorded SBP, DBP, and HR during implant placement surgeries in order to compare the values obtained at the beginning of the intervention with the maximum values. In a manner comparable to other studies [14,18], these authors observed significant differences between the values recorded, confirming an increase in BP during surgery. They point out the relevance of these data and the need to control BP, especially in patients who already have high baseline values.

The study by Balasubramaniyan et al. [20] was conducted on a sample of hypertensive patients attending a university clinic for dental extractions. They recorded the pre-anesthesia dental anxiety level using the APAIS (Amsterdam Preoperative dental anxiety and Information Scale). Again, after anesthesia, they recorded SBP, DBP, and HR at 5, 10, and 15 min. Twenty percent of the patients in the severe dental anxiety group had a significant increase in SBP and HR compared to the mild dental anxiety group.

Patients were divided into normotensive and hypertensive groups as previously published studies [8,9]. Olmo-Gonzalez et al. [8] sought to determine whether there were alterations in SBP and DBP or HR fluctuations in normotensive versus hypertensive participants during surgical and nonsurgical dental treatments and using anesthetic with or without vasoconstrictor. They included 200 participants over 65 years of age. Five recording points were established, and treatments were performed with and without anesthesia. Significant differences were observed in the evolution of the SBP, with an initial increase in participants who underwent surgical treatment and in those who did not receive a vasoconstrictor.

When analyzing the differences in BP and HR between patients with different levels of dental anxiety, we found a very large group of patients with low dental anxiety and very small groups in the rest of the levels. For this reason, it was decided to group the patients into a group with less than 9 points on the Corah Dental Anxiety Scale and a group with 9 or more points on this scale. Both SBP and DBP and HR were higher in patients with a higher level of dental anxiety, although the differences were only significant in HR. These data are consistent with studies previously published by Silvestre et al. [9] and Fernandez-Aguilar [7].

As expected, periodontal treatments were the ones in which patients registered the highest levels of dental anxiety. These are long procedures, especially if performed by students; periodontal probing is always unpleasant, especially in patients with periodontal pathology. In addition, they also require radiographs. Its positioning in the mouth, the number of repetitions on certain occasions, and the variation in the opening depending on the patient often make it a non-pleasant moment, and therefore, a higher level of dental anxiety is registered. Needless to say, patients with greater dental anxiety would have more periodontal problems because they visit the dentist less frequently. Periodontal re-evaluation is conducted one month after the mechanical treatment and maintenance every six months. This assumes that the patient knows exactly what he/she is facing, and the memory he/she has of the previous treatment would influence the score obtained in the questionnaire. As expected, revisions scored lower than the first visits (6 [IQR 3] and 5 [IQR 2] points, respectively). When a patient goes to the dental office for the first time, they do not know which professional will attend them, they do not know the facilities or the protocols followed in the same, and this makes them feel more anxious. This is especially true for patients who have not been to the dentist for a long time and who assume that they will have a lot of dental pathologies. On the other hand, when they go for a check-up in a known place, they feel comfortable because they are in an environment that is familiar to them, and they know the protocols and the way the students work.

### Limitations and Strengths of the Study

This study only included patients who were seen in a university clinic. In the future, it would be desirable to be able to carry out a multicentric study, including patients from different places and in different cities. In addition, it would be interesting to introduce another variable to check whether the level of dental anxiety is related to socioeconomic level.

Likewise, this work has focused on the assessment of dental anxiety, but the sample is composed of very few patients with high levels of dental anxiety. Obtaining a sample with a higher dental anxiety level would allow the study of these patients and possible correlations with physiological parameters.

Another limitation to address is the heterogeneity of the sample regarding treatments, and the difference between first, second, and other visits later on.

There are numerous scales [21,22,23] that record the level of dental anxiety. Using different instruments for the measurement of dental anxiety in addition to the Corah Dental Anxiety Scale would allow the results obtained with each of them to be compared. Likewise, they could be completed at different times throughout the treatment in order to be able to monitor dental anxiety at the different points of the dental intervention.

Finally, including different surgical and non-surgical treatments with and without local anesthesia would make it possible to compare the effect of these treatments on dental anxiety, BP, and HR with respect to treatments without local anesthesia.

## 5. Conclusions

In this group of patients, no significant correlations were found between the levels of dental anxiety and blood pressure either in the patients as a whole or when hypertensive and normotensive patients were studied separately during the noninvasive dental treatments. With regard to heart rate and duration of the treatments, significant, although weak, correlations were found between the level of dental anxiety and heart rate and between the level of dental anxiety and the duration of the treatments in the patients as a whole and in the normotensive patients, but not in the hypertensive patients.

As for the evolution of physiological parameters over time, all patients showed a progressive decrease in values during treatment. In hypertensive patients, systolic blood pressure and heart rate also decreased significantly. Diastolic blood pressure did not show these differences. In normotensive patients, a significant decrease in all parameters was observed.

The present study aims to establish a roadmap for the dentist and the responsibility of intervening and reducing the state of dental anxiety that visits the dental office generate in patients. To begin with, the practitioner should consider the psychological condition of the patient and thus adapt as far as possible to the patient’s needs. Although the health professional is an authority figure, it is important to listen to the patient, especially during the first visit, to know his past experiences and expectations so that he feels that the person who is going to treat him is in agreement with him. In this way, we will be able to reduce this psychological stress and therefore avoid episodes of arterial hypertension that pose a great risk to patients.

## Figures and Tables

**Table 1:** Values of SBP, DBP, and HR for all patients (*n* = 200).

	Median [IQR]
**SBP (mmHg)**	
At arrival	126.5 [32.5]
3 min before	121.5 [30]
3 min after	124 [32]
At the end	123 [29]
**DBP (mmHg)**	
At arrival	80 [16]
3 min before	78 [17]
3 min after	79 [18]
At the end	79 [19]
**HR (bpm)**	
At arrival	74 [16.5]
3 min before	75 [17.5]
3 min after	72 [17]
At the end	70 [5]

SBP: systolic blood pressure; DBP: diastolic blood pressure; HR: heart rate; IQR: interquartile range.

**Table 2:** Values of SBP, DBP, and HR for the hypertensive (*n* = 48) and normotensive (*n* = 152) groups.

	Hypertensive	Normotensive	
	Median [IQR]	Median [IQR]	*p*-Value
**SBP (mmHg)**			
At arrival	142.5 [26]	120 [30]	**<0.001**
3 min before	139.5 [33]	118 [27.5]	**<0.001**
3 min after	144.5 [28]	118.5 [26]	**<0.001**
At the end	139.5 [26.5]	119 [31]	**<0.001**
**DBP (mmHg)**			
At arrival	86.5 [15]	77 [14.5]	**<0.001**
3 min before	85 [29]	75 [14.5]	**<0.001**
3 min after	85 [18]	76 [16]	**<0.001**
At the end	84 [14]	77 [18]	**<0.001**
**HR (bpm)**			
At arrival	73 [15.5]	74 [17]	0.603
3 min before	71 [14]	76 [17.5]	0.080
3 min after	70 [11.5]	74 [18]	0.124
At the end	66 [13.5]	71 [14.5]	**0.021**

SBP: systolic blood pressure; DBP: diastolic blood pressure; HR: heart rate; IQR: interquartile range.

**Table 3:** Correlations between the Corah Dental Anxiety Scale score and HR and time of treatment.

	All (*n* = 200)		Hypertensive (*n* = 48)		Normotensive (*n* = 152)	
VARIABLE	*Spearman’s rho*	*p-Value*	*Spearman’s rho*	*p-Value*	*Spearman’s rho*	*p-Value*
HR at arrival	0.128	0.072	0.132	0.373	0.128	0.116
HR 3 min before	**0.166**	**0.019**	0.217	0.140	0.155	0.057
HR 3 min after	**0.176**	**0.013**	0.244	0.095	**0.166**	**0.041**
HR at the end	0.120	0.090	0.220	0.134	0.091	0.265
Time of treatment	**0.191**	**0.007**	0.198	0.198	**0.190**	**0.020**

HR: heart rate.

**Table 4:** Comparisons of study variables between sexes (Median [IQR]).

Variables	Group of Patients (*n* = 200)			Normotensive (*n* = 152)			Hypertensive (*n* = 48)		
	Male (*n* = 75)	Female (*n* = 125)	*p*-Value	Male (*n* = 47)	Female (*n* = 105)	*p*-Value	Male (*n* = 28)	Female (*n* = 20)	*p*-Value
Dental anxiety (score)	4 [2]	6 [2]	**0.001**	4 [2]	6 [1]	**<0.001**	4 [3]	7 [3.5]	**0.025**
SBP at arrival (mmHg)	139 [27]	119 [28]	**0.001**	130 [33]	115 [25]	**<0.001**	145.5 [24]	138 [27.5]	0.086
DBP at arrival (mmHg)	85 [18]	77 [12]	**0.001**	81 [16]	76 [12]	0.075	90.5 [12.5]	83 [12.5]	0.057
HR at arrival (bpm)	72 [21]	75 [15]	0.196	72 [21]	75 [15]	0.464	72 [17]	76 [16]	0.250

SBP: systolic blood pressure; DBP: diastolic blood pressure; HR: heart rate; IQR: interquartile range.

**Table 5:** Comparisons of study variables by age (Median [IQR]).

Variables	Group of Patients (*n* = 200)			Normotensive (*n* = 152)			Hypertensive (*n* = 48)		
	≥52 years (*n* = 102)	<52 years (*n* = 98)	*p*-Value	≥52 years (*n* = 61)	<52 years (*n* = 91)	*p*-Value	≥52 years (*n* = 41)	<52 years (*n* = 7)	*p*-Value
Dental anxiety (score)	5 [3]	6 [3]	0.464	5 [3]	6 [3]	0.473	4 [3]	4 [11]	0.613
SBP at arrival (mmHg)	139.5 [27]	115.5 [26]	**<0.001**	131 [34]	113 [25]	**<0.001**	142 [22]	155 [53]	0.474
DBP at arrival (mmHg)	83 [17]	76 [13]	**<0.001**	79 [16]	75 [12]	**0.031**	86 [13]	89 [31]	0.501
HR at arrival (bpm)	73 [16]	74 [16]	0.422	74 [15]	74 [16]	0.622	73 [16]	78 [15]	0.715

SBP: systolic blood pressure; DBP: diastolic blood pressure; HR: heart rate; IQR: interquartile range.

**Table 6:** Comparisons of study variables by type of treatment (Median [IQR]).

Variables	Prothesis (*n* = 29)	Periodontal Studies (*n* = 19)	First Visits (*n* = 74)	Prophylaxis (*n* = 29)	Check-Up (*n* = 49)	*p*-Value
Dental anxiety (score)	4 [2]	7 [4]	6 [3]	4 [4]	5 [2]	**0.006**
SBP at arrival (mmHg)	128 [31]	135 [35]	127 [29]	132 [30]	117 [40]	0.349
DBP at arrival (mmHg)	83 [9]	86 [23]	80 [16]	82 [16]	75 [14]	0.118
HR at arrival (bpm)	74 [16]	73 [16]	77.5 [19]	72 [16]	72 [14]	0.407

SBP: systolic blood pressure; DBP: diastolic blood pressure; HR: heart rate; IQR: interquartile range.

**Table 7:** Analysis of physiological parameter values between patients with different levels of anxiety.

	Low Dental Anxiety (<9 Points) (*n* = 173)	Moderate-High Dental Anxiety (≥9 Points) (*n* = 27)	
	Median [IQR]	Median [IQR]	*p*-Value
SBP (mmHg)	126 [34]	131 [31]	0.941
DBP (mm Hg)	79 [16]	80 [19]	0.713
HR (bpm)	73 [16]	81 [24]	**0.032**

SBP: systolic blood pressure; DBP: diastolic blood pressure; HR: heart rate; IQR: interquartile range.

## Data Availability

Not applicable.
